# Supplementary material for: Benefits of Maternal Choline Supplementation on Aged Basal Forebrain Cholinergic Neurons (BFCNs) in a Mouse Model of Down Syndrome and Alzheimer’s Disease
Source: Biomolecules. 2025 Aug 5;15(8):1131. doi: 10.3390/biom15081131 (PMC12384390; doi:10.3390/biom15081131)
Supplement: Supplementary file 1 [file biomolecules-15-01131-s001.zip › Supplementary Text_Alldred_biomolecules.pdf]

Figure S1. Schematic representation of bioinformatics workflow, including pre-processing and downstream analysis.

Figure S2. **A**. Normalized cell counts are presented as mean values in heatmap, comparing disomic (2N), disomic MCS (2N+), trisomic (Ts) and trisomic MCS (Ts+) for excitatory neurons (ExcN), inhibitory neurons (InhN), astrocytes (Astro), oligodendrocytes (Oligo), oligodendrocyte progenitor cells (OPC), microglia (MG), endothelial cells (Endo) and T-cells (T). All conditions were significant comparing ExcN to each of the other 7 cell types ($p<0.83E-5$ or lower). Within ExcN, group differences were not significant (ns). Bar graphs indicate upregulation and downregulation of significant genes in the disomic diet (2N+ versus 2N; **B**), supplemented genotype (Ts+ versus 2N+; **C**) and diet plus genotype (Ts+ versus 2N; **D**). Volcano plots show fewer highly significant DEGs in disomic diet (**E**) compared to supplemented genotype (**F**) and diet plus genotype (**G**).

Figure S3. GO analysis examined the relationship between unique disease diet processes, MCS responsive processes and unique genotype processes are shown for each category. The *y*-axis indicates relative percentage of total processes per category with disease diet (teal), MCS responsive (green), and genotype (magenta).

Figure S4. Aging and MCS responsiveness in trisomic mice. **A**. Genotype dependent pathways were altered by aging and MCS, although few pathways show rescue from ~6 MO to ~11 MO. Grey line differentiates ~11 MO rescue and additive selected pathways. **B**. Select D/Fs were rescued by MCS regardless of age, however, some D/Fs were genotype, MCS or age dependent as seen by heatmap of selected processes.

Table S1. DEGs by genotype in ~11 MO BFCNs.

Table S2. DEGs by disease diet comparison in ~11 MO BFCNs.

Table S3. DEGs by disomic diet comparison in ~11 MO BFCNs.

Table S4. DEGs by supplemented diet comparison in ~11 MO BFCNs.

Table S5 DEGs by diet plus genotype comparison in ~11 MO BFCNs.

Table S6. IPA analysis of significant neuronal canonical pathways by genotype in ~11 MO BFCNs.

Table S7. IPA analysis of significant neuronal canonical pathways by disease diet comparison in ~11 MO BFCNs.

Table S8. IPA analysis of significant D/Fs by genotype in 11 ~11 MO BFCNs.

Table S9. IPA analysis of significant D/Fs by disease diet comparison in ~11 MO BFCNs.

Table S10. GO analysis of significant processes by genotype in 11 ~11 MO BFCNs.

Table S11. GO analysis of significant processes by disease diet comparison in ~11 MO BFCNs.
