# Supplementary figures and images for: Benefits of Maternal Choline Supplementation on Aged Basal Forebrain Cholinergic Neurons (BFCNs) in a Mouse Model of Down Syndrome and Alzheimer’s Disease
Source: Biomolecules. 2025 Aug 5;15(8):1131. doi: 10.3390/biom15081131 (PMC12384390; doi:10.3390/biom15081131)

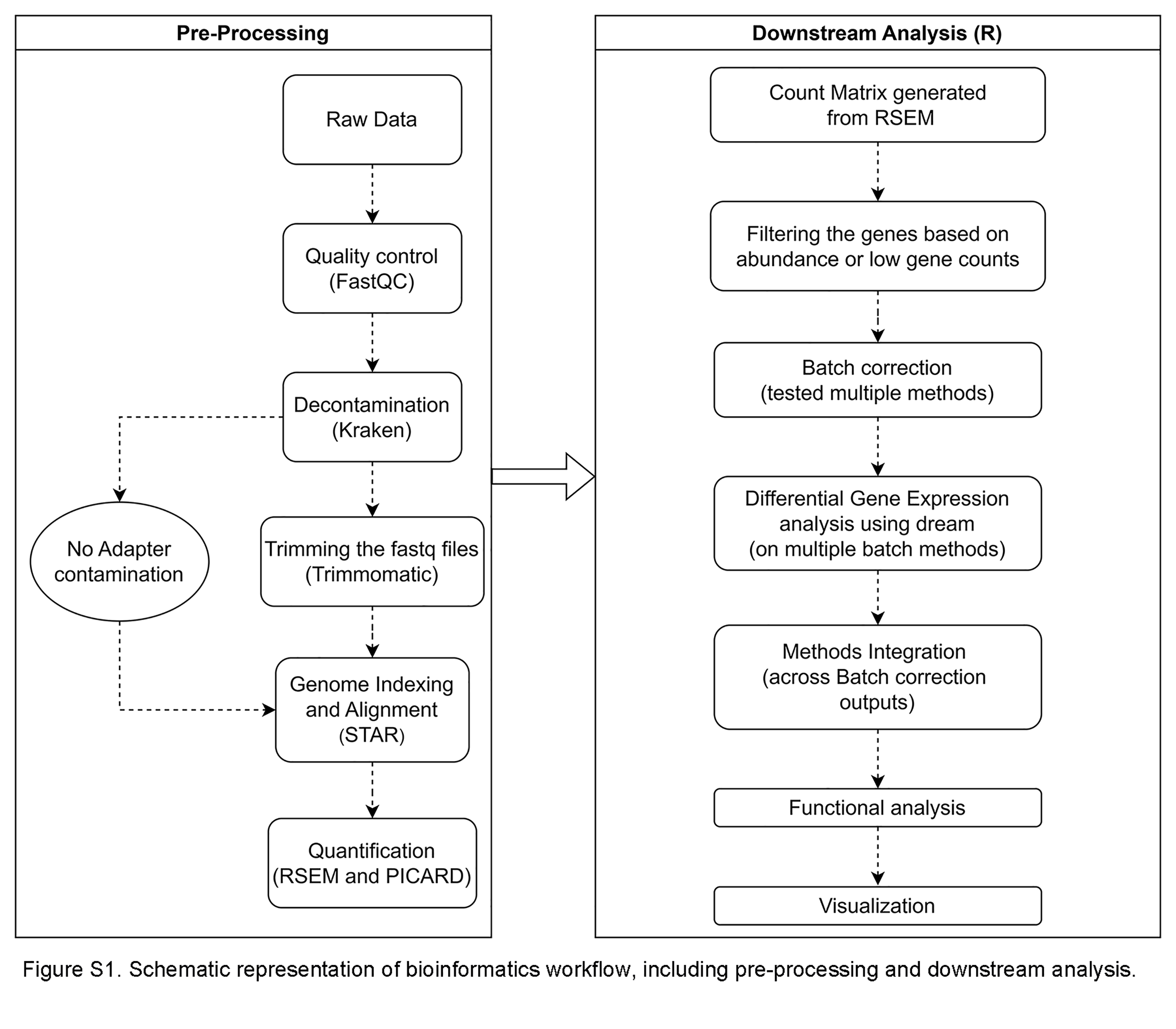

Supplement: Supplementary file 1 [file biomolecules-15-01131-s001.zip › Figure S1_final.tif]

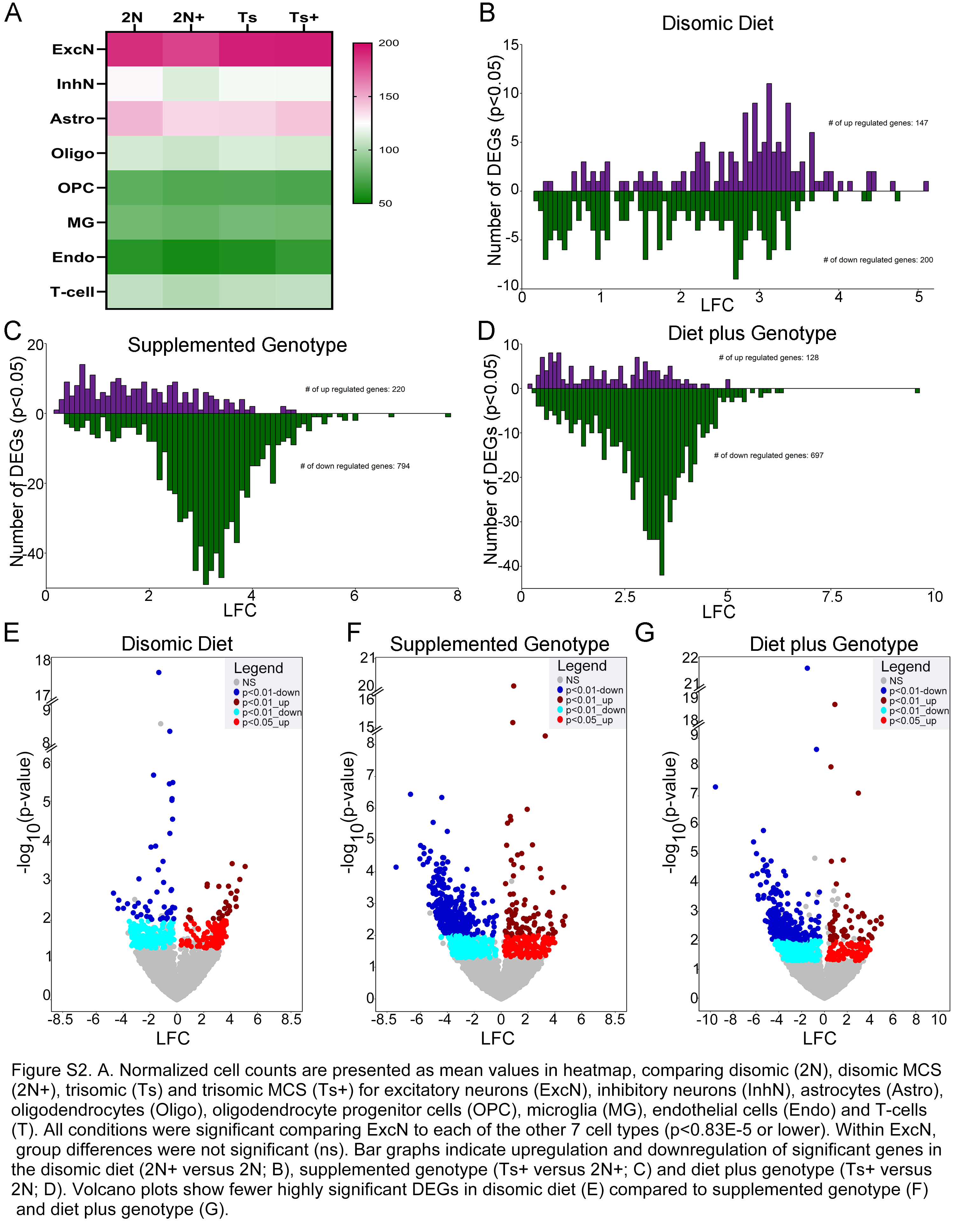

Supplement: Supplementary file 1 [file biomolecules-15-01131-s001.zip › Figure S2.final.tif]

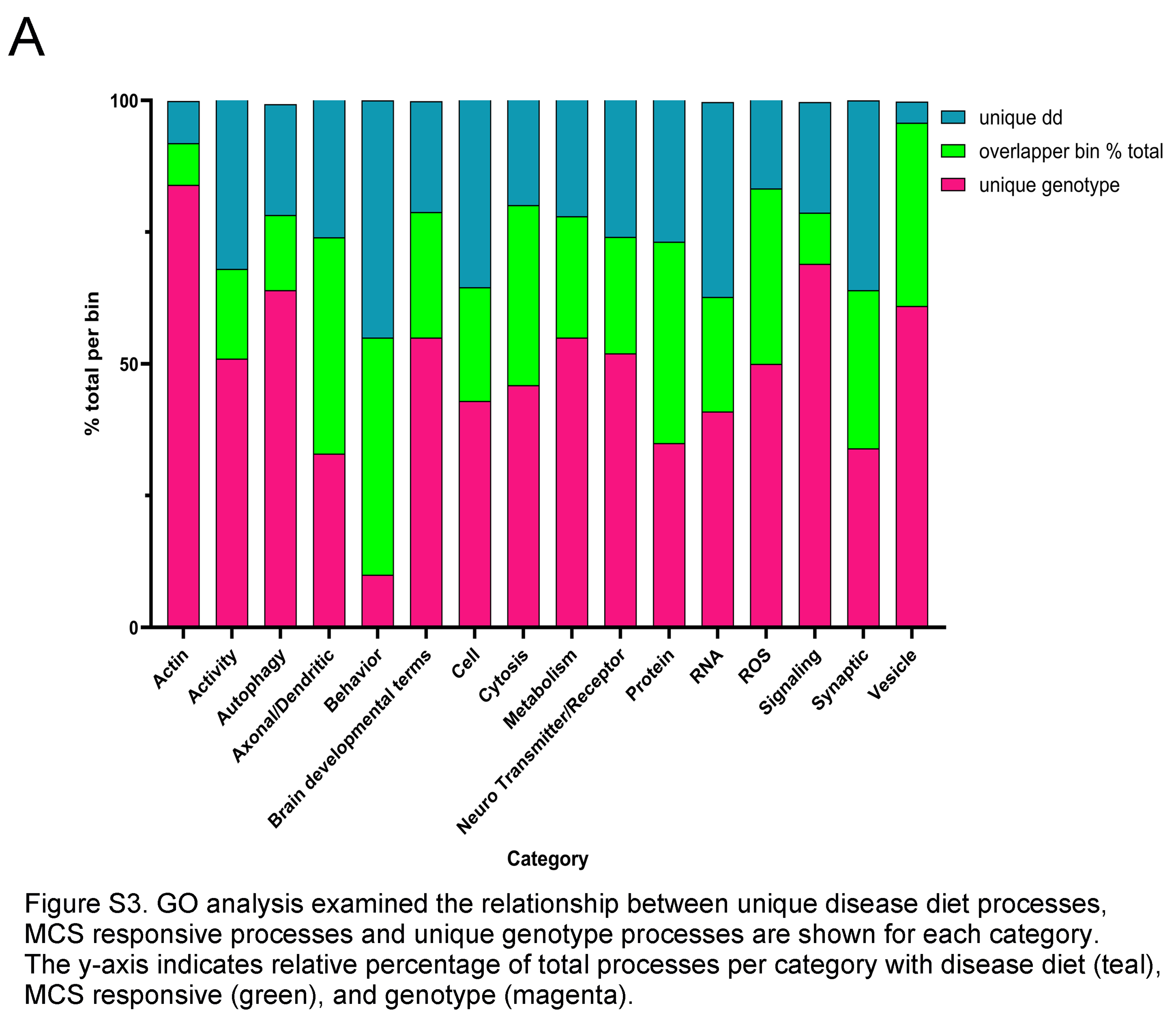

Supplement: Supplementary file 1 [file biomolecules-15-01131-s001.zip › Figure S3_final.tif]

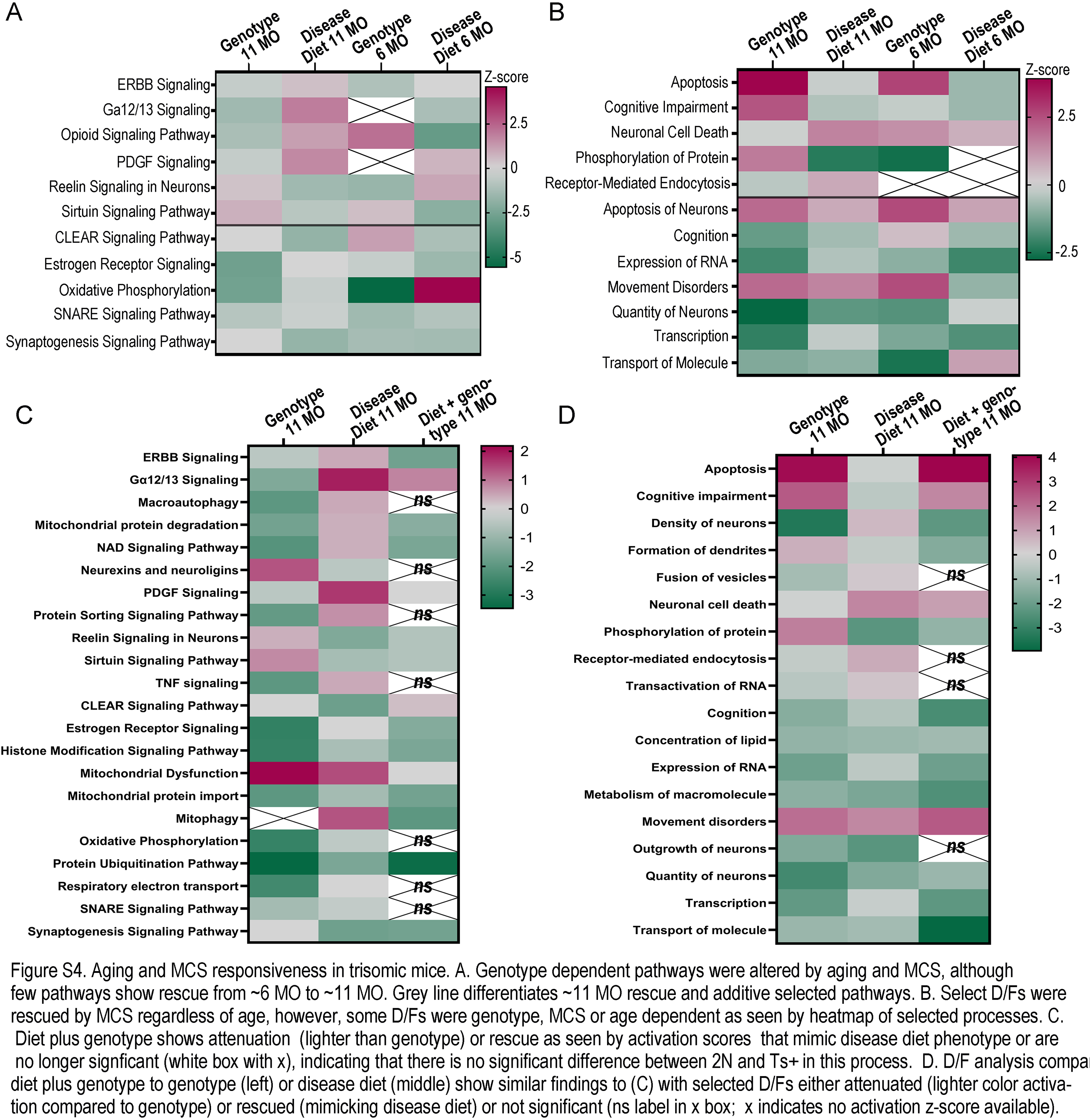

Supplement: Supplementary file 1 [file biomolecules-15-01131-s001.zip › Figure S4_final_resized_R1.tif]
